# An Appropriate Modulation of Lymphoproliferative Response and Cytokine Release as Possible Contributors to Longevity

**DOI:** 10.3390/ijms18071598

**Published:** 2017-07-24

**Authors:** Irene Martínez de Toda, Carmen Vida, Mónica De la Fuente

**Affiliations:** 1 Department of Animal Physiology II, Faculty of Biology, Complutense University, Madrid 28040, Spain; irene_mc90@hotmail.com (I.M.d.T.); carmenvidarueda@hotmail.com (C.V.); 2 Institute of Investigation Hospital 12 Octubre, Madrid 28041, Spain

**Keywords:** proliferation, cytokine, aging, longevity, leukocytes

## Abstract

The decrease in the proliferative response of lymphocytes is one of the most evident among the age-related changes of the immune system. This has been linked to a higher risk of mortality in both humans and experimental animals. However, long-lived individuals, in spite of optimally maintaining most of the functions of the immune system, also seem to show an impaired proliferative response. Thus, it was hypothesized that these individuals may have distinct evolution times in this proliferation and a different modulatory capacity through their cytokine release profiles. An individualized longitudinal study was performed on female ICR-CD1 mice, starting at the adult age (40 weeks old), analyzing the proliferation of peritoneal leukocytes at different ages in both basal conditions and in the presence of the mitogen Concanavalin A, for 4, 24 and 48 h of culture. The cytokine secretions (IL-2, IL-17, IL-1β, IL-6, TNF-α and IL-10) in the same cultures were also studied. Long-lived mice show a high proliferative capacity after short incubation times and, despite experiencing a functional decline when they are old, are able to compensate this decrease with an appropriate modulation of the lymphoproliferative response and cytokine release. This could explain their elevated resistance to infections and high longevity.

## 1. Introduction

It is known that the aging process is accompanied by a deterioration of the competence of the immune system. Thus, with aging there is an increased susceptibility to infectious diseases, autoimmune and cancer processes, which exerts a great influence on age-related morbidity and mortality [1,2]. Aging of the immune system, which is known as immunosenescence, involves a striking rearrangement of almost every component, leading to changes including enhanced as well as diminished functions [3,4]. In addition, the functioning of the immune system has been demonstrated to be an excellent marker of health, given that several age-related changes in immune functions are predictive of mortality and lifespan [3,5,6,7,8,9]. Thus, long-lived individuals seem to exhibit a high degree of preservation of several functions of the immune system with values similar to those observed in adult individuals. This may be essential to reach a very advanced age in a healthy condition [3,10,11,12,13,14].

Among all the age-related changes that the immune system undergoes, the most obvious is the involution of the thymus gland [15,16]. Accordingly, one of the most marked age-related alterations in the immune cells has been reported in the T lymphocytes, specifically in the lymphoproliferative response to mitogens, which is decreased in old subjects for both humans and experimental animals [13,14,17,18,19]. The study of the proliferative response of leukocytes to a given stimulus has become an important issue given that a low lymphoproliferative response to mitogens has been linked to an increased mortality, and together with other parameters, defines the immune risk phenotype in humans [20,21]. Moreover, this low lymphoproliferative response reported in human peripheral blood cells has also been found in peritoneal, spleen, lymphoid nodes and thymus leukocytes from rodents, and it is related to a higher morbidity and mortality in aged animals [3,9,14]. However, there are conflicting results regarding this function in long-lived individuals, given that most of the studies describe a similar response to that of older people [22,23].

Cytokines are principal mediators of interactions among immune cells. They are responsible for the development and resolution of immune response and are greatly affected by the aging process [3,7,24,25,26]. In fact, an age-related loss of homeostasis in cytokine networks can contribute significantly to health impairment in old age [26]. In this context, together with the previously mentioned age-related loss of functionality in immune cells, aging is characterized by a chronic low-grade inflammatory status, so-called “inflamm-aging” [7,25]. Thus, it has been described that an age-related increase in release of pro-inflammatory cytokines in resting cells leads to a sterile inflammation [4]. This is accompanied by an elevation of circulating levels of cytokines in old subjects, such as IL-6, which in addition has been related to a higher risk of mortality [7,27,28,29]. However, cells from old subjects produce lower pro-inflammatory cytokines when needed to do so, i.e., after a mitogenic stimulus, compared to those of adult subjects [24,30,31]. Again, long-lived individuals, despite having high levels of pro-inflammatory markers, have a postponed disease onset, making it difficult to understand whether “inflamm-aging” is beneficial or detrimental [32].

Based on the striking facts regarding lymphoproliferation and cytokine release by immune cells in long-lived individuals, it was hypothesized that these individuals could present different proliferative as well as cytokine release dynamics as an adaptive mechanism [10,33]. Moreover, given that all the studies in long-lived individuals previously mentioned have been cross-sectional, it is still not known if they reach those advanced ages due to the maintenance of optimal immune cell function during their whole life (as if they were adults) or whether they experience an age-related impairment in these functions but are able to compensate for it.

In order to address these questions, an individualized longitudinal study was performed on female ICR-CD1 mice analyzing the proliferation as well as the cytokine secretion profile of leukocytes obtained from peritoneum of animals at different ages. The study was performed starting at the adult age, 40 weeks old, and followed each animal individually until its death. The proliferative capacity as well as the cytokine release profile was studied in both un-stimulated/basal conditions and after incubation with a T-cell mitogenic stimulus (Concanavalin A) for 4, 24 and 48 h.

## 2. Results

**Figure 1:**
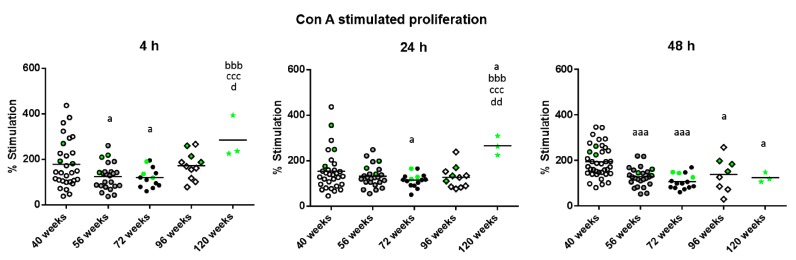
Stimulation of proliferation, expressed as percentages, in response to Con A (1 μg/mL), of peritoneal leukocytes from adult (40 weeks), mature (56 weeks), old (72 weeks), very old (96 weeks) and long-lived (120 weeks) mice after 4, 24 and 48 h of culture. The green points represent the values in mice that reached 120 weeks. a: *p* < 0.05; aaa: *p* < 0.001 with respect to the value in adult mice. bbb: *p* < 0.001 with respect to the value in mature mice. ccc: *p* < 0.001 with respect to the value in old mice. d: *p* < 0.05; dd: *p* < 0.01 with respect to the value in very old mice.

The results of the lymphoproliferation after incubation with the T-cell mitogenic stimulus Concanavalin A (Con A), expressed as the percentage of stimulation, are shown in Figure 1. There was a decreased proliferation in mature mice (56 weeks of age) compared to when they were adults (40 weeks of age) after 4 h (*p* < 0.05) and 48 h of incubation (*p* < 0.001). At old age (72 weeks of age), they also had decreased proliferation compared to when they were adults after 4, 24 (*p* < 0.05) and 48 h of incubation (*p* < 0.001). The decrease in this proliferation in cells of very old mice (96 weeks of age) with respect to when they were adults, was only observed at 48 h of incubation (*p* < 0.05). However, when mice reached high longevity (120 weeks of age) they showed an increased proliferation with respect to when they were mature, old and very old at 4 and 24 h (*p* < 0.001, *p* < 0.05 and *p* < 0.01, respectively). With respect to when they were adults, the percentage of proliferation only increased at 24 h (*p* < 0.05) whereas at 48 h they showed a decrease (*p* < 0.05). The green points represent the values of mice that reached 120 weeks of age, and it can be observed that the three long-lived mice always show values above the mean.

**Figure 2:**
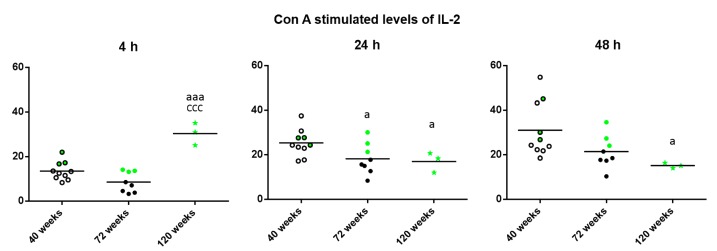
Levels (pg/mL) of IL-2 releases by peritoneal leukocytes from adult (40 weeks), old (72 weeks) and long-lived (120 weeks) mice, after 4, 24 and 48 h of culture in presence of Con A (1 μg/mL). The green points represent the values in mice that reached 120 weeks. a: *p* < 0.05; aaa: *p* < 0.001 with respect to the value in adult mice. ccc: *p* < 0.001 with respect to the value in old mice.

Regarding the levels of IL-2 released in the cultures in presence of Con A (Figure 2), striking differences depending on the incubation time were found. Thus, after 4 h, increased levels were observed in the long-lived mice with respect to when they were adult and old (*p* < 0.001). However, after 24 h of incubation, long-lived mice as well as old mice showed decreased levels compared to when they were adult (*p* < 0.05) and after 48 h of incubation, long-lived mice showed decreased levels compared to when they were adult (*p* < 0.05). It can be observed that in general, the three long-lived mice (highlighted in green) show values above the mean of the group.

**Figure 3:**
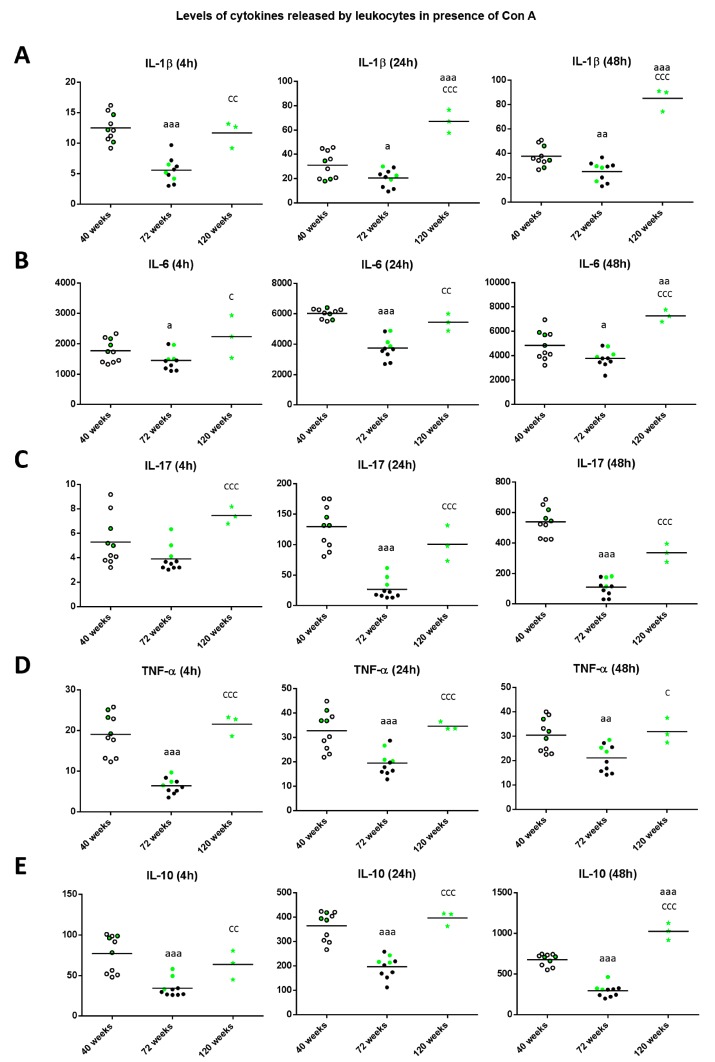
Levels (pg/mL) of IL-1β (**A**); IL-6 (**B**); IL-17 (**C**); TNF-α (**D**) and IL-10 (**E**) released by peritoneal leukocytes of adult (40 weeks), old (72 weeks) and long-lived (120 weeks) mice, after 4, 24 and 48 h of culture in presence of Con A (1 μg/mL). The green points represent the values in mice that reached 120 weeks. a: *p* < 0.05; aa: *p* < 0.01; aaa: *p* < 0.001 with respect to the value in adult mice. c: *p* < 0.05; cc: *p* < 0.01; ccc: *p* < 0.001 with respect to the value in old mice.

With respect to the levels of cytokine released in the cultures in presence of Con A (Figure 3), the levels of IL-1β (Figure 3A) decreased at old age in comparison to those when the animals were adult (*p* < 0.001 after 4 h, *p* < 0.05 after 24 h, *p* < 0.01 after 48 h of culture). However, mice reaching high longevity showed an increase in the levels of this cytokine with respect to when they were old (*p* < 0.01 at 4 h and *p* < 0.001 at 24 and 48 h of culture) and similar to when they were adults at 4 h of culture or even higher (*p* < 0.001) at 24 and 48 h. Regarding the levels of IL-6 (Figure 3B), again at old age they decreased in comparison to those when the animals were adults (*p* < 0.05 after 4 and 48 h; *p* < 0.001 after 24 h of culture), whereas when mice reached high longevity these levels increased with respect to when they were old (*p* < 0.05, *p* < 0.01 and *p* < 0.001 after 4, 24 and 48 h, respectively), being similar to the levels obtained when they were adults (at 4 and 24 h) or higher (*p* < 0.01 at 48 h). The results obtained with IL-17 (Figure 3C), show that old mice presented decreased levels with respect to when they were adult after 24 and 48 h of incubation (*p* < 0.001), whereas long-lived mice showed increased levels with respect to when they were old (*p* < 0.001) independently of the incubation time. Thus, long-lived mice showed similar levels to when they were adults.

With respect to the levels of TNF-α (Figure 3D), old mice showed decreases compared to when they were adult (*p* <0.001 at 4 and 24 h, *p* < 0.01 at 48 h of culture), whereas long-lived mice showed increased levels with respect to when they were old (*p* < 0.001 after 4 and 24 h, and *p* < 0.05 after 48 h) and similar to when they were adults. Regarding the levels of the anti-inflammatory cytokine IL-10 (Figure 3E), old mice showed decreased values in comparison to those when they were adults (*p* < 0.001 independently of the incubation time). However, long-lived mice showed increased levels with respect to when they were old (*p* < 0.01 after 4 h, *p* < 0.001 after 24 and 48 h) and similar to when they were adults (at 4 and 24 h of culture) and even increased (*p* < 0.001) to when they were adults after 48 h of culture. It can be observed that in general, the three long-lived mice (highlighted in green) always show values in all these cytokines above the mean when they were adult and old animals.

**Figure 4:**
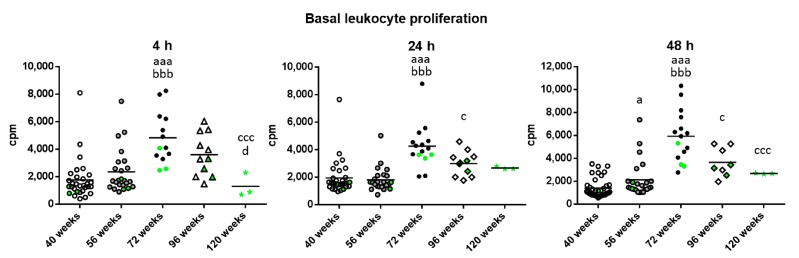
Basal proliferation (cpm) of peritoneal leukocytes from adult (40 weeks), mature (56 weeks), old (72 weeks), very old (96 weeks) and long-lived (120 weeks) mice, after 4, 24 and 48 h of culture. The green points represent the values in mice that reached 120 weeks. a: *p* < 0.05; aaa: *p* < 0.001 with respect to the value in adult mice. bbb: *p* < 0.001 with respect to the value in mature mice. c: *p* < 0.05; ccc: *p* < 0.001 with respect to the value in old mice. d: *p* < 0.05 with respect to the value in very old mice.

With respect to basal proliferation (Figure 4), which is what takes place in the absence of a proliferative stimulus, there was a significant increase in the old mice with respect to when they were adult and mature (*p* < 0.001) independently of the incubation time. Furthermore, there was a decrease in very old mice compared to when they were old after 24 and 48 h of incubation (*p* < 0.05). This decrease was also found in long-lived compared to old mice at 4 and 48 h (*p* < 0.001) and to very old mice at 4 h (*p* < 0.05). However, very old and long-lived mice showed similar levels to when they were adults and mature. It can be observed that the three long-lived mice always show values below the mean for basal proliferation.

**Figure 5:**
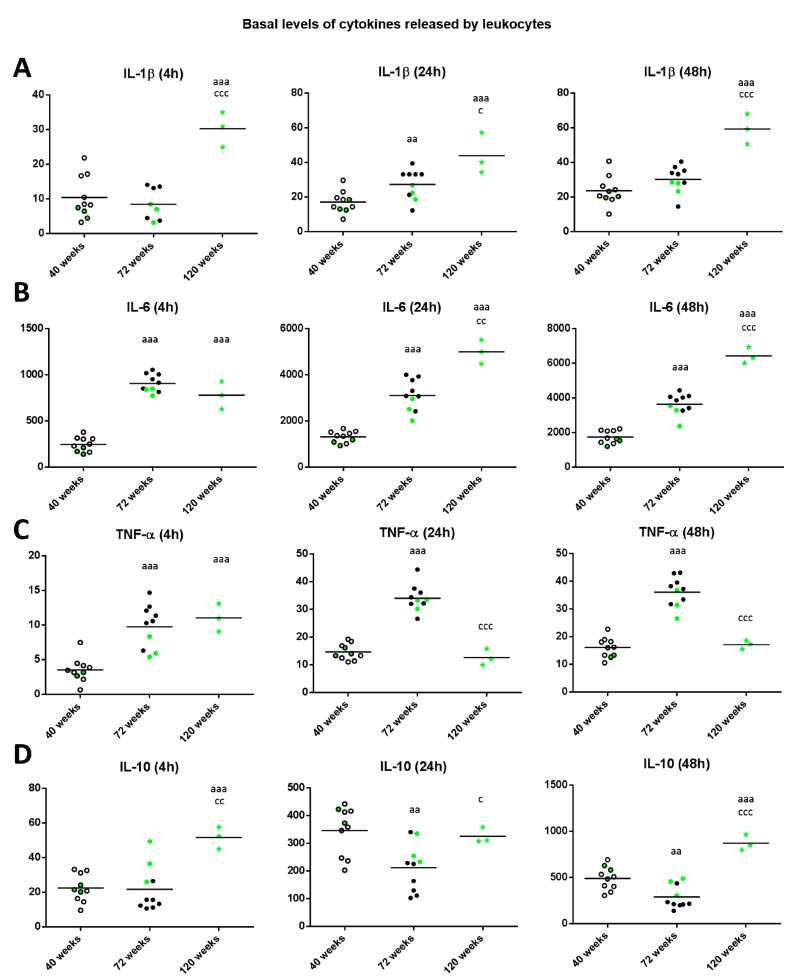
Levels (pg/mL) of IL-1β (**A**); IL-6 (**B**); IL-17 (**C**); TNF-α (**D**) and IL-10 (**E**) released by peritoneal leukocytes from adult (40 weeks), old (72 weeks) and long-lived (120 weeks) mice after 4, 24 and 48 h of culture. The green points represent the values in mice that reached 120 weeks. aa: *p* < 0.01; aaa: *p* < 0.001 with respect to the value in adult mice. c: *p* < 0.05; cc: *p* < 0.01; ccc: *p* < 0.001 with respect to the value in old mice.

Regarding the levels of IL-1β released in basal conditions (Figure 5A), old mice showed increased levels compared to when they were adults after 24 h of incubation (*p* < 0.01). Moreover, there was a significant increase in long-lived mice with respect to when they were adult independently of the incubation time (*p* < 0.001) and with respect to when they were old (*p* < 0.05 after 24 h, *p* < 0.001 after 4 and 48 h). The levels of IL-6 in basal conditions (Figure 5B), in both old and long-lived mice showed increased levels compared to when they were adults (*p* < 0.001 independently of the incubation time). Moreover, there was a significant increase in long-lived mice with respect to when they were old (*p* < 0.01 after 24 h, *p* < 0.001 after 48 h of culture).

Regarding the levels of TNF-α in basal conditions (Figure 5C), old and long-lived mice showed increased levels (*p* < 0.001) compared to when they were adults after 4 h of incubation. In addition, after 24 and 48 h of incubation, old mice showed increased levels compared to when they were adults (*p* < 0.001), whereas long-lived mice showed decreased levels (*p* < 0.001) with respect to those when they were old and similar to when they were adults.

With respect to IL-10 levels in basal conditions (Figure 5D), old mice showed decreased levels compared to when they were adults (*p* < 0.01 after 24 and 48 h of culture). Moreover, in long-lived mice the levels were similar (24 h of culture) or increased (*p* < 0.001 after 4 and 48 h of incubation) with respect to those when they were adults, and with respect to when they were old (*p* < 0.01 after 4 h, *p* < 0.05 after 24 h and *p* < 0.001 after 48 h). It can be observed that in general, the three long-lived mice (highlighted in green) always show values below the mean for the proinflammatory cytokines IL-1β, IL-6y TNF-α when they were adult and old, whereas show values above the mean for the anti-inflammatory cytokine IL-10.

**Figure 6:**
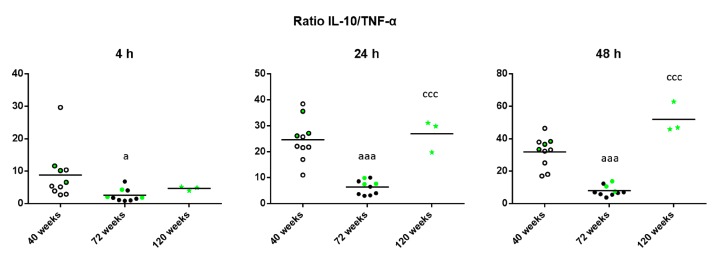
IL-10/TNF-α ratios of the levels of these cytokines released by peritoneal leukocytes from adult (40 weeks), old (72 weeks) and long-lived (120 weeks) mice after 4, 24 and 48 h of culture. The green points represent the values in mice that reached 120 weeks. a: *p* < 0.05; aaa: *p* < 0.001 with respect to the value in adult mice. ccc: *p* < 0.001 with respect to the value in old mice.

Finally, given that the maintenance of health relies on the adequate balance of antiinflammatory and proinflammatory mediators, the IL-10/TNF-α ratio was calculated in unstimulated conditions to shed light on the age-related changes of this balance. As shown in Figure 6, old mice have a decreased IL-10/TNF-α ratio compared to when they were adults (*p* < 0.05 after 4 h, *p* < 0.001 after 24 and 48 h of culture). However, long-lived mice show the opposite results, with an increased IL-10/TNF-α ratio compared to when they were old (*p* < 0.001 after 24 and 48 h).

## 3. Discussion

This is the first study which analyzes the age-related changes in lymphoproliferation as well as in cytokine release under resting and stimulated conditions at different incubation times in a longitudinal study, monitoring individually each animal throughout their lifetime.

Lymphocytes are important regulator and effector cells in adaptive immunity, and their activation and proliferation is essential for an appropriate immune response and, consequently, to the maintenance of homeostasis. The age-related decrease in Con A-induced proliferation shown in the present work, which mimics the stimulation by antigens [34], is in agreement with the results obtained from many previous studies carried out in 48 h cultures [13,14,17,19,35,36,37]. However, this is the first work showing that at 4 and 24 h of culture, lymphocytes from old mice also present a decrease in their percentage of proliferative stimulation.

It has been suggested that oxidative stress impairs the ability of lymphocytes to respond to a stimulus such as mitogenic or antigenic stimuli [38,39,40], so the age-related increase in oxidative stress that is known to occur in immune cells [41,42], could be the underlying mechanism for the age-related decrease in Con A-induced proliferation. Another possible explanation could be that given that immune cells replicate multiple times due to chronic stimulation or encountering many pathogens during a lifetime, they lose their proliferation capacity and may reach the stage of replicative senescence [43,44]. However, cells of mice achieving high longevity maintain this capacity but with different dynamics. Thus, there was a stronger proliferative response in these animals after 4 and 24 h of incubation with Con A than when they were younger. Furthermore, after 48 h of incubation these differences were no longer noticeable. Since the results are shown individually, it is possible to observe that those mice that achieved a high longevity, and presented proliferative values above the mean of the age group throughout their lives. Moreover, the strong increase of proliferation at 4 and 24 h in these animals could suggest a faster capacity of the immune response against antigens, which may represent an immune advantage. A possible explanation for the fact that long-lived mice show less proliferation after 48 h of incubation than after 4 and 24 h could be due to an increase in the activation-induced cell death (AICD) after 24 h. AICD plays a key role in the maintenance of immune system homeostasis and it is of physiological importance, as the presence of too many activated cells might trigger excessive secondary immune responses leading to symptoms similar to autoimmune disorders and toxic shock [45,46,47,48]. Another explanation for the increased lymphoproliferative response at 4 h in long-lived mice could be the higher release of IL-2, which occurs at that time. In fact, IL-2 is of great importance for proliferative T-cell response [49] and it is well-established that aging is related to an impaired release of IL-2 by in vitro stimulated T cells in rodents and humans [13,49]. In the present study, the lower IL-2 levels at 24 and 48 h of culture found in old mice in comparison to their adult values are in agreement with all previous studies.

It is known that immune cells communicate with each other through cytokines, which are soluble mediators that, through a complex network of interactions, are crucial for the functioning of the immune system and its fine-tuning. With aging, the production, release and action of these cytokines suffer impairments [3,7,24,25,26]. In this context, most of the studies of immunosenescence have focused on isolated subsets of immune cell types [26] and on the levels of cytokines after 48 h of culture [24]. The data described here are thus of special relevance, since this study was performed using unfractionated peritoneal leukocytes in order to reproduce more accurately the in vivo cytokine response. Furthermore, the age-related changes in cytokine release were analyzed after different incubation times. Thus, in presence of Con A, old mice show a decreased secretion of pro-inflammatory cytokines (IL-1β, IL-6 and IL-17) and anti-inflammatory cytokines (IL-10) compared to when they were adults, in agreement with previous results obtained in cross-sectional studies on mice [24]. The release of such cytokines is of great importance for the triggering of the immune response. Thus, IL-1β is an important mediator in infection that stimulates intrinsic, innate and adaptive immune pathways [50] as well as IL-6, which contributes to host defense through the stimulation of acute phase responses, hematopoiesis and immune reactions [51]. In addition, IL-17 has been recently described as an important orchestrator of immunity [52]. Thus, the reduced release of these cytokines in stimulated leukocytes from old mice could be the cause of the impaired immune responses found in both aged humans and rodents [3,13,14,20,21,53]. In mice that achieve a high longevity, the values of these cytokines, which were above the media of the age group throughout their lives, were, in many cases, similar to those when they were adults. These results agree with those obtained in cross-sectional studies previously carried out [24]. The data obtained in these cross-sectional studies could make one think that those animals that reach high longevity, maintain an immune function similar to adults during their lifetime. However, the results of the present study demonstrate that animals which reach high longevity experience an age-related decrease of cytokine release capacity in response to a stimulus when they are old, but they possess better compensatory mechanisms which allow for its recovery when they are long-lived. A possible explanation for the fact that long-lived mice show even higher levels of pro-inflammatory cytokines in some cases compared to when they were adults or old will be discussed later in this article.

Regarding what is known about the age-related changes in the lymphoproliferative response, most of the research performed has been focused on the ability of lymphocytes to proliferate towards a stimulus, whereas there are almost no studies that have investigated the proliferative ability of lymphocytes in unstimulated/basal conditions. However, the study of lymphoproliferation in basal conditions could be a useful age-related marker, given that it provides information on how much inner damage the immune system is trying to fight. It is known that damage-associated molecular patterns (DAMPs) increase with the advance of age due to the larger number of tissue injuries in the body and that these molecules are able to trigger an activation of the immune system response by initiating the release of pro-inflammatory cytokines [54,55]. In the present study, it has been found that old mice exhibit a significant increase in the basal proliferation with respect to when they were younger, whereas long-lived mice show basal proliferative levels similar to when they were adults. The high proliferation in the absence of stimulus seen in old mice implies a deregulation of the immune system. This suggests that peritoneal leukocytes from old mice are overactivated, possibly due to DAMPs, which, through the release of cytokines, could be responsible for the establishment of “sterile inflammation” in the elderly [3,54]. In addition, it has been suggested that oxidative-inflammatory stress can activate signaling pathways that lead to proliferation, which would also explain this proliferation of lymphocytes without antigen stimulation [40,56,57]. In fact, in the present study, old mice showed a higher basal release of pro-inflammatory cytokines, such as IL-1β, IL-6 and TNF-α, compared to when they were adults. This higher basal release could be responsible for the establishment of the chronic low-grade inflammation that is known to occur in old individuals. Notably, long-lived mice showed even higher basal release of pro-inflammatory cytokines, such as IL-1β and IL-6, compared to when they were old. This fact is striking given that high levels of IL-6 and IL-1β have been referred to as the most powerful predictors of morbidity and mortality in the elderly [28,29] and naturally long-lived mice would be expected to have withstood the detrimental effects of the aging process better than those individuals who do not live to extreme old age. Thus, these high levels of pro-inflammatory cytokines could be the consequence of the successful adaptation to a number of stresses, including infections, which unceasingly occur throughout life, as other authors have proposed [4,58]. Even though low-grade chronic inflammation is a characteristic of aged people and centenarians, it has been suggested that the difference between them is that the long-lived are able to avoid the main age-related diseases and reach exceptional ages due to the contrasting action of anti-inflammatory agents [59,60]. In the present study, it was shown that leukocytes from long-lived mice release high levels of the anti-inflammatory cytokine IL-10 in unstimulated conditions, even higher than when they were adult, which could be the underlying mechanism for the maintenance of health in longevity, as it has also been found in long-lived rats [61]. In addition, the high release of IL-10 in leukocytes of long-lived mice after 4 h of culture can explain the decrease in TNF-α levels found in these animals at 24 and 48 h of incubation, since IL-10 inhibits the release of TNF-α [62,63]. Moreover, the present study demonstrated that the balance between anti-inflammatory and pro-inflammatory mediators (IL-10/TNF-α ratio), could be a useful parameter, given that its values are similar in adult and long-lived mice, whereas old mice show a decreased ratio. In fact, a recent study pointed at the anti-inflammatory/inflammatory ratio as an indicator of successful aging and longevity [61].

Altogether, it seems that the chronic basal pro-inflammatory state shown by non-stimulated leukocytes from old animals in the present work is linked to a defective inflammatory response upon stimulation that will ultimately limit the ability of these animals to deal with infections [54]. In fact, 85-year-old humans who produce low ex vivo levels of Lipopolysaccharide-induced pro- and/or anti-inflammatory cytokines, such as IL-6, TNF-α and IL-10, have more than a twofold higher mortality risk compared to their age-matched counterparts with a higher cytokine production [64]. Conversely, extremely long-lived mice in general showed a similar cytokine profile upon Con A stimulation to the one when they were adults, demonstrating that immune cells from long-lived individuals have achieved the capacity to trigger an adequate immune response. It is crucial to bear in mind that the study of the age-related changes of both lymphoproliferation as well as cytokine release at only one point in time may not be optimal [65]. The results of the present study are in agreement with this idea due to the finding that long-lived mice have an earlier response both in lymphoproliferation as well as in cytokine release compared to when they were old.

Another significant contribution of the study is the determination of the age-related changes regarding lymphoproliferative and cytokine release ability, which a long-lived individual experiences throughout its lifetime, from its adulthood until its death. Thus, the present study reveals that those mice that naturally achieve high longevity are the ones that not only maintained lower levels of basal proliferation and higher levels of proliferation after Con A stimulation during their whole lifetime, but are also those that achieve a better control of the effects of aging on the immune functions. Thus, long-lived mice are those that maintained a lower secretion of pro-inflammatory cytokines and a higher secretion of anti-inflammatory cytokines in unstimulated conditions as well as a higher one upon Con A-stimulation when they were old, compared to their age-matched counterparts. Moreover, this is the first study to demonstrate that the animals reaching high longevity experience immune-senescent changes (to a lesser extent than those which do not reach advanced ages), but they are able to compensate for them by showing optimal levels when they are long-lived. According to a perspective recently suggested [66], the best candidates to become long-lived are not the strongest and most robust subjects among their age cohort, but subjects that better adapt to the environment, showing more biological plasticity. Thus, we observed in the present study that if the effects of age are suffered by all individuals, those that exhibited better capacity of control of the situation by improving the release of the anti-inflammatory cytokine IL-10 and restraining the release of pro-inflammatory cytokines such as TNF-α, achieved higher longevity. Furthermore, the study suggests that the IL-10/TNF-α ratio in unstimulated conditions is a better indicator of mouse longevity than any of the inflammatory mediators solely. More studies are needed to corroborate if this good control capacity in long-lived individuals could be shown in other immune cell responses.

## 4. Materials and Methods

### 4.1. Animals

Female ICR/CD1 ex-reproductive mice (Mus musculus) were used in this study. Animals were purchased from Janvier Labs (Aachen, Germany) at the adult age (32 ± 4 weeks), and were placed and acclimatized in the Animal Facility at the Faculty of Biology of Complutense, University of Madrid (UCM) (Spain). Mice were housed at 4–5 per cage and maintained in standard laboratory animal conditions for pathogens, temperature (22 ± 2 °C) and humidity (50–60%), on a 12/12 h reversed light/dark cycle (lights on at 20:00 h) to avoid circadian interferences. Mice had access to tap water and standard pellets (Panlab, Barcelona, Spain) ad libitum. One group of animals (*n* = 40) was used for the longitudinal study. The collection of peritoneal suspensions was at the adult (40 ± 4 weeks; *n* = 38), mature (56 ± 4 weeks; *n* = 25), old (72 ± 4 weeks; *n* = 15), very old (96 ± 4 weeks; *n* = 8) and long-lived (120 ± 4 weeks; *n* = 3) ages. All the animals had a natural death and whereas no weight loss (<20%), moribund state or tumor formation were detected, the cause of death was not investigated further. All the experiments were approved by the Experimental Animal Committee of Complutense, University of Madrid (UCM) (Spain) and were in accordance to the guidelines of the European Community Council Directives 2010/63/EU of 22 September 2010.

### 4.2. Collection of Peritoneal Leukocytes

Peritoneal suspensions containing unfractioned leukocytes, were collected from each mouse between 08:00 and 10:00 h to minimize circadian variations of the immune parameters studied without killing the animals, which allowed a longitudinal study to be performed. Without using anesthesia, mice were held by cervical skin and 3 mL of sterile Hank’s solution at 37 °C was injected into the peritoneum. After massaging the abdomen of the mouse, approximately 80% of the injected volume, containing the peritoneal leukocytes, was recovered using the needle employed for the Hank´s injection. Leukocytes from peritoneal cell suspensions were quantified in Neubauer chambers using optical microscopy (40×). Cell viability was checked by the Trypan Blue exclusion test and only cell suspensions with cell viability of 99% or higher were used.

### 4.3. Lymphoproliferation

The proliferation capacity of lymphocytes was evaluated by the method previously described [67]. Peritoneal cell suspensions were adjusted to 5 × 10^5^ lymphocytes/mL in complete medium containing RPMI-1640, 10% fetal bovine serum and 1% gentamicin. 200 µL containing 1 × 10^5^ lymphocytes were dispensed into 96-well plates. 20 µL/well of complete medium or Con A (1 μg/mL), a T cell mitogen lectin, were added, for basal and stimulated conditions, respectively. The plates were incubated at 37 °C in a sterile and humidified atmosphere of 5% CO_2_ for 4, 24 and 48 h. After these time points, 100 µL of culture supernatants were collected for cytokine measurements and afterwards, 2.5 µCi ^3^H-thymidine (Hartmann Analytic, Braunschweig, Germany) together with 100 µL of fresh medium were added to each well, followed by another incubation of 24 h. Cells were harvested in a semiautomatic harvester (Skatron Instruments, Tranby, Norway) and thymidine uptake was measured in a β counter (LKB, Upsala, Sweden) for 1 min. The results were calculated as ^3^H-thymidine uptake (counts per minute, cpm) for basal and stimulated conditions, and basal proliferation was expressed as cpm whereas stimulated proliferation was expressed as the percentage of lymphoproliferation capacity (%) giving the value 100 to the cpm in basal conditions.

### 4.4. Cytokine Measurement

In order to study the levels of cytokines in the environment surrounding immune cells influencing the lymphoproliferative responses, supernatant samples were collected after each incubation time (4, 24 and 48 h) in the absence or presence of Con A. For this, the samples were obtained from the same culture well-plates used for determining proliferation prior to the addition of ^3^H-thymidine. In aliquots of 100 µL of culture supernatants, the levels of cytokines, including growth factors (IL-2), proinflammatory cytokines (IL-1β, IL-6, TNF-α and IL-17) as well as the anti-inflammatory cytokine IL-10, were measured simultaneously by multiplex luminometry (Beadlyte mouse multiplex cytokine detection system, MHSTCMAG-70K, Millipore, Billerica, MA, USA). The measurements were carried out in samples of adult, old and long-lived mice.

### 4.5. Statistical Analysis

SPSS 21.0 (SPSS, Chicago, IL, USA) was used for the statistical analysis of the results. All data are expressed as the mean of the values corresponding to subjects, each value being the mean of duplicate assays. The normality of the samples and the homogeneity of variances were checked by the Kolmogorov–Smirnov and Levene analyses, respectively. Differences due to age were studied through Student´s *t*-test for independent samples. Two-sided *p* < 0.05 was considered the minimum level of significance.

## Abbreviations

AICD	Activation-induced cell death
Con A	Concanavalin A
DAMPs	Damage associated molecular patterns
LPS	Lipopolysaccharide
